# Mason bees and honey bees synergistically enhance fruit set in sweet cherry orchards

**DOI:** 10.1002/ece3.10289

**Published:** 2023-07-09

**Authors:** Julia Osterman, Frances Benton, Sara Hellström, Meike Luderer‐Pflimpfl, Ann‐Kathrin Pöpel‐Eisenbrandt, Bilyana Stoykova Wild, Panagiotis Theodorou, Christin Ulbricht, Robert J. Paxton

**Affiliations:** ^1^ General Zoology, Institute for Biology Martin‐Luther‐University of Halle‐Wittenberg Halle (Saale) Germany; ^2^ Department of Computational Landscape Ecology Helmholtz Centre for Environmental Research‐UFZ Leipzig, ESCALATE Leipzig Germany; ^3^ Nature Conservation and Landscape Ecology, Faculty of Environment and Natural Resources University of Freiburg Freiburg Germany; ^4^ Gothenburg Global Biodiversity Centre University of Gothenburg Göteborg Sweden; ^5^ Queen's University Belfast Belfast UK; ^6^ Service Verband Gartenbau Thüringen e.V. Erfurt Germany; ^7^ Faculty of Biology Sofia University “St. Kliment Ohridski” Sofia Bulgaria; ^8^ German Centre for Integrative Biodiversity Research (iDiv) Halle‐Jena‐Leipzig Leipzig Germany; ^9^ Dezernat Gartenbau Landesanstalt für Landwirtschaft und Gartenbau Quedlinburg Germany

**Keywords:** *Apis mellifera*, crop pollination, ecological intensification, European orchard bee, *Osmia cornuta*, pollination management, stocking density

## Abstract

Mason bees (*Osmia* spp.) are efficient fruit tree pollinators that can be encouraged to occupy and breed in artificial nesting material. In sweet cherry orchards, they are occasionally used as an alternative managed pollinator as a replacement for or in addition to honey bees (*Apis mellifera*). Yet, the lack of practical guidelines on management practices, for example optimal stocking rates, for both mason bee nesting material and honey bees might compromise pollination service provision. In this study, we assessed the relationship between stocking rates (honey bee hives and mason bee nesting material) and the abundance of honey bees and mason bees in 17 sweet cherry (*Prunus avium*) orchards in Central Germany. We furthermore performed a pollination experiment to explore the interactive effect of mason bees and honey bees on sweet cherry fruit set. In the orchards, both honey bee and mason bee abundance increased with increasing stocking rates of hives or nesting material, respectively. Honey bee abundance increased linearly with stocking rates. In contrast, mason bee abundance asymptoted at 2–3 nesting boxes per ha, beyond which more boxes resulted in little increase in visitation rate. Our pollination experiment demonstrated that orchards were pollen limited, with only 28% of insect‐pollinated flowers setting fruit versus 39% of optimally hand‐pollinated flowers. Honey bees and mason bees enhanced sweet cherry fruit set, but only when both were present and not when either was present alone in an orchard. Our findings demonstrate that offering nesting material for mason bees and employing honey bee hives can enhance bee abundance in sweet cherry orchards. By increasing honey bee abundance in combination with enhanced mason bee abundance, farmers can substantially boost fruit set and potentially sweet cherry yield. To enhance pollination services, farmers should consider the benefits of increasing pollinator biodiversity as an immediate benefit to improve crop yields.

## Graphical abstract

We present data from our field experiment carried out in 17 cherry orchards in Central Germany. We found that the number of honey bees and mason bees can be enhanced through proving honey bees hives and artificial nesting material. However, sweet cherry fruit set only increased when both species were enhanced, suggesting a synergistic effect of bee species on fruit set.

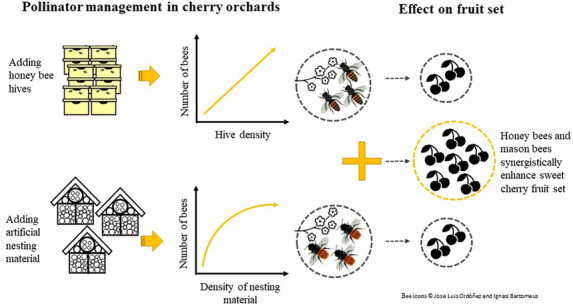

## 1 INTRODUCTION

For many crops, insect pollination is essential for successful fruit development (Klein et al., 2007), including sweet cherry (*Prunus avium* (L.) Moench). Most sweet cherry cultivars are self‐sterile and require cross‐pollination (Free, 1993), to which wild pollinators are thought to contribute significantly (Eeraerts et al., 2017, 2019a; Holzschuh et al., 2012). However, habitat loss and land‐use intensification due to agriculture are among various human pressures that have contributed to declines in wild pollinator abundance and diversity (Dicks et al., 2021; Millard et al., 2021; Potts et al., 2016). Intensive cherry fruit cultivation in Belgium is linked to a reduction in pollinator species richness and abundance, subsequently decreasing sweet cherry fruit set (Eeraerts et al., 2017). Likewise, with the loss of wild bee habitat in the landscape surrounding German cherry orchards, visitation rates of wild bees have been shown to decrease along with fruit set and yield (Holzschuh et al., 2012). Reduced fruit set of sweet cherry has also been documented in North America, possibly due to a lack of pollinators (Reilly et al., 2020).

In intensified agricultural landscapes with little natural habitat, farmers can actively engage in pollinator management or enhance local bee habitats on farms to ensure sufficient provision of crop pollination (Garibaldi et al., 2017; Osterman, Aizen, et al., 2021). Worldwide, 66 species of insect can be managed for pollination, of which the western honey bee (*Apis mellifera* L.) is the most prominent (Osterman, Aizen, et al., 2021) and most frequently employed by farmers (Breeze et al., 2019; Osterman, Landaverde‐González, et al., 2021). Mason bees (*Osmia* spp.) have also been used as managed pollinators for many decades, especially for the pollination of rosaceous fruit trees, including cherry (Bosch & Kemp, 2001, 2002; Güler, 2020; Hamroud et al., 2022; Kornmilch, 2010; Krunic et al., 1991; Maeta & Kitamura, 1974; Torchio, 1976).

The acceptance by some mason bees of multiple artificial nesting materials (e.g. wooden blocks, bamboo, cardboard nesting material) and their gregarious nesting behaviour are important preconditions for their successful mass rearing and feasible species management (Torchio, 1976). Moreover, mason bees have several traits that make them a suitable pollinator for sweet cherry cultivars that flower in early spring, a time when inclement weather spells are frequent that could lead to subsequent negative effects on fruit set (Roversi & Ughini, 1996). The flight activity of honey bees is severely restricted at ambient temperatures below 12°C, limiting their effective pollination of an early flowering crop such as sweet cherry (Vicens & Bosch, 2000a). Mason bee species, in contrast, maintain their flight activity under low ambient temperatures, in light rain and during windy conditions, ensuring a more uniform and consistent pollination service that is largely independent of inclement weather (Bosch & Kemp, 1999; Vicens & Bosch, 2000b). *Osmia cornuta* (Latreille, 1805), the European orchard bee, is one of the first bees to emerge in spring in Central Europe, followed by *O. bicornis* (red mason bee; Linnaeus, 1758; Megachilidae) (Eeraerts et al., 2021; Westrich, 2018); the phenology of both, but especially *O. cornuta*, coincides with the flowering of sweet cherry, reinforcing their potential role in sweet cherry pollination. Mason bees are also considered effective pollinators of sweet cherry flowers due to their putative higher efficiency (Eeraerts, Vanderhaegen, et al., 2019). Their higher rate of row changes compared to honey bees or bumble bees potentially ensures good pollen transfer for a crop like sweet cherry (Eeraerts, Vanderhaegen, et al., 2019; Mateos‐Fierro et al., 2022) that requires cross‐pollination to set fruit.

Despite the long history of mason bee management, in some regions only honey bees are used as managed pollinators in sweet cherry orchards (Eeraerts et al., 2020). One reason might be a lack of evidence‐based practical guidelines for crop‐specific pollination management, including spatial configuration of nesting material for solitary bees (Bosch et al., 2021). Even optimal honey bee management guidelines are missing for many crops (Mallinger et al., 2021; Rollin & Garibaldi, 2019), which could result in diminished crop yields or elevated costs of pollination management.

The contribution of mason bees to fruit set has been assessed in several crops (Boyle & Pitts‐Singer, 2019; Pitts‐Singer et al., 2018; Ryder et al., 2019; Sheffield, 2014), but their effect on sweet cherry pollination is little understood. Interestingly, the results of a recent meta‐data analysis suggest positive effects of *Osmia* spp. management on pollination when employed in combination with *Apis*, but not when employed alone (Hünicken et al., 2022). One reason for this could be that the presence of non‐*Apis* bees, which include mason bees, has been shown to alter the foraging behaviour of honey bees by causing them to switch trees more frequently within an orchard, making them more efficient pollinators and leading to higher fruit set or yields (Brittain et al., 2013; Eeraerts et al., 2019b; Greenleaf & Kremen, 2006; Pitts‐Singer et al., 2018; Sapir et al., 2017). Yet, the interactive effect of bee species on fruit set is rarely studied (Brittain et al., 2013; Hünicken et al., 2022; Sapir et al., 2017).

In this study, we aimed to test (1) if mason bee and honey bee populations can be enhanced through the provision of nesting material and employing hives, respectively, by assessing their abundance in 17 commercial sweet cherry (*P. avium*) orchards in Germany that vary in intensity of habitat enhancement for wild bees. We furthermore tested (2) whether *Osmia* spp. and honey bees synergistically increase sweet cherry fruit set by assessing the relationship between the abundance of mason bees and honey bees in relation to fruit set across orchards. Based on our results, we give recommendations for farmers on pollination management to ensure stable fruit set in sweet cherry orchards.

## 2 METHODS

### 2.1 Study sites

**FIGURE 1:**
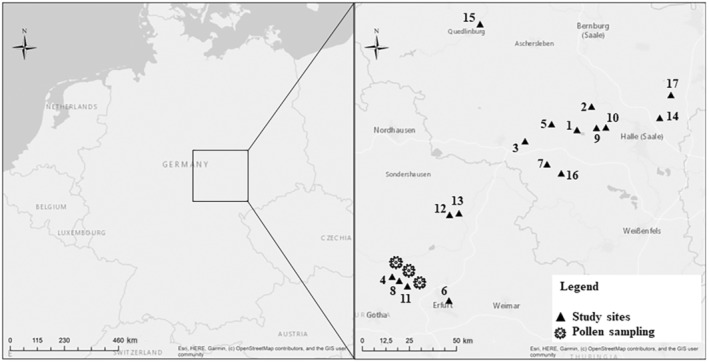
Study sites in Central Germany. In 2020, bee abundance and fruit set were measured in 17 sweet cherry orchards (black triangles) with varying bee management. In a subset of three sites (sites 4, 8 and 11, labelled with an additional pollen grain icon), trap nests for mason bees were installed and the identity pollen in the provisioned nests was examined.

Fieldwork was carried out in spring 2020 in orchards within the German federal states of Sachsen‐Anhalt and Thuringia (Figure 1). Both federal states are dominated by agriculture (>60% of land cover). We selected 17 sites, of which two were experimental orchards and 15 were commercial mixed fruit orchards (Table S1). The experimental orchards are owned by research institutions for conducting experiments but are managed as commercial orchards in order to simulate agriculturally field‐realistic conditions. The size of the orchards devoted to sweet cherry (*P. avium*) cultivation ranged between 0.2 and 36 ha (6.6 ± 8.6; mean ± SD). Distances between field sites were >2 km (47.9 ± 28.9 km; mean ± SD) to ensure spatial independence.

Pollination management consisted of providing honey bee hives and nesting material for mason bees, specifically *O. cornuta*. Though *O. bicornis* is common in the study region, its flight period only commences after cherry has ceased to flower and therefore we did not consider it further in this study. Commercial bumble bee nests were not used by cherry farmers in this study. To investigate the effect of bee management on bee abundance and subsequently on sweet cherry fruit set, we selected sites with varying pollinator management. Mason bee management ranged from 0 (i.e. no nesting material provided) to 8.6 nesting boxes per ha. On‐farm bee management was decided prior to the study by the farmer and therefore the size of the nesting boxes varied across sites. To make them comparable, we set the standard size of a nesting box to be 100 × 54 cm and containing approximately 500 tubes made of paper, bamboo or drilled wood, each of a length of 13–16 cm and a diameter of 4–10 mm (Figure S1). Farmers initiated mason bee management in different years, ranging from 2002 to 2020 (Table S1). In the two sites at which farmers started providing nesting material in the same year as the study (2020), *O. cornuta* cocoons were provided in addition to the nesting material to ensure similar conditions (Table S1). The number of honey bee colonies per site (Table S1) varied from 0 to 20 hives/ha.

### 2.2 Flower‐visitor observations

We quantified the abundance of insect flower visitors during the peak bloom of sweet cherry (16.04.2020–23.04.2020) at each site for 1 day. For each site, two transect walks of 90 min each (in total 180 min) were performed alongside cherry trees on a sunny day, one in the morning and one in the afternoon on the same day. Transect walks were performed >50 m from the edge of the orchard and included trees of the cultivar ‘Regina’ used for a pollination experiment (described below). One observer walked continuously alongside the ‘Regina’ cultivar while noting all visible flower visitors. Flower visitors that touched the reproductive parts of a cherry flower were counted and identified to morphological group: honey bees, bumble bees, mason bees (*O. cornuta*), other wild bees, butterflies, flies, beetles and ants. Though flower‐visitor observations performed only for 1 day might be a limiting factor, assessments in cherries with a very limited flowering period of around 5–8 days restrict observations over several days in multiple orchards. Ambient (shade) temperatures during the transect walks were recorded with a digital thermometer; they ranged from 9 to 22°C.

### 2.3 Verification of mason bees as cherry flower visitors

To confirm that managed mason bee populations are flower visitors of cherries, we identified the source of the pollen with which *O. cornuta* females provisioned their nests. To do so, we distributed 30 cardboard nesting tubes (15 of 7 mm diameter and 15 of 9 mm diameter) per site at three sites (Figure 1; sites 4, 8 and 11) before cherry bloom (end of February/beginning of March). These 30 cardboard nesting tubes were located on top of the diverse nesting boxes that had already been provided by orchardists. During full cherry bloom (22.04.2020), the tubes were collected, cut open and the pollen provisions of the larvae were extracted. Per site, we created a pooled pollen sample, which was later divided into three samples per site; pollen identification of each pool was performed by the Hohen Neuendorf Länderinstitut für Bienenkunde (https://www2.hu‐berlin.de/bienenkunde/). A solution was made of one volume of pollen and four volumes of water. Drops of this solution were applied to a microscope slide, dried and then fixed with glycerol‐gelatine (Kearns & Inouye, 1993). Under a microscope, all pollen grains were identified and quantified by counting 500 grains per pooled sample following the DIN‐Norm 10760, which is a standardised protocol to determine the relative frequency of pollen from different plant taxa in honey samples. Pollen grains were identified to genus level where possible but in one case only to family level (Ranunculaceae).

### 2.4 Pollination service provisioning in cherry orchards

In order to quantify provision of pollination services, we studied one of the most common sweet cherry cultivars, ‘Regina’, which was present at all 17 study sites. This cultivar is self‐sterile (S‐alleles: S_1_S_3_) and requires cross‐pollination for successful fruit development (Holzschuh et al., 2012; Lech et al., 2008). We chose at least one row of ‘Regina’ trees in each orchard, which was planted either next to a cross‐compatible pollinizer cultivar or which was interspersed with a pollinizer cultivar in the same row. Pollinizer cultivars varied across sites (Table S1). We selected 20 ‘Regina’ trees in each orchard that were at least 50 m from the orchard edge. On each tree, we applied three flower treatments: insect exclusion (‘bagged’: B), hand pollination (‘hand’: H) and open insect pollination (‘open’: O). For each treatment, we chose a flower bundle, which we marked with coloured ribbons, cord, and barrier tape to later locate the treatments. In some cases, several flower bundles were used for one treatment if one flower bundle contained less than three flowers. During full bloom and on the same day as observations of flower visitors, we counted all open and receptive flowers per bundle and removed old (over‐flowered) or young (still closed) flowers. The insect exclusion treatment (B) was bagged in fine netting (1 mm PVC mesh) prior to cherry bloom (6.04.2020–12.04.2020) to prevent insect pollination and remained bagged throughout cherry bloom; it enabled us to estimate the contribution of wind pollination and autonomous self‐pollination to fruit set. Flower bundles of treatment H were manually pollinated with pollen from at least two flowers of an adjacent pollinizer (see Table S1 for pollinizer cultivars per site) as a measure of maximal fruit set. Treatment O remained unmanipulated as a measure of current pollination service provision at each fruit orchard; O treatment flowers were accessible to all flower‐visiting insects including honey bees and mason bees.

Sweet cherry fruit set was counted three times, once in May to assess initial fruit set (ca. 4 weeks after flowering), once in June after the so‐called June fall (ca. 8 weeks after flowering), and once immediately prior to harvest to assess final fruit set, approximately in the beginning of July (ca. 12 weeks after the experimental manipulations). We divided the number of developed fruits per bundle by the number of flowers per bundle for each fruit count period (i.e. May, June and July) to give the percentage fruit set per treatment. In addition, we recorded the weight of each cherry fruit during the final fruit set count in July.

### 2.5 Statistical analysis

All data were analysed in R version 4.1.2 (R Core Team, 2022). A Spearman rank correlation test was used to check the independence of honey bee hive management and the density of mason bee nesting material. To assess the effect of nesting material density and honey bee hive density on the abundance of mason bees and honey bees, respectively, as flower visitors of sweet cherry, we used generalised linear mixed models (GLMMs) using the *lme4* package (Bates et al., 2017). In both models we included ambient temperature as an additional predictor variable to test its impact on insect abundance. Location (orchard) was included as a random factor. As honey bees have been shown to suppress wild bee abundance (Herbertsson et al., 2016; Lindström et al., 2016; Weekers et al., 2022), we included honey bee abundance as a fixed factor for predicting mason bee abundance. We compared linear models with non‐linear models reaching an asymptote (*y* ~ log (*x* + 1)) by using the Akaike information criterion corrected for small sample size (AICc) as we expected a saturation effect of pollinator management on flower‐visitor abundance. The best fit model was chosen by ΔAICc > 2 with the *AICc* function in the *MuMIn* package (Bartoń, 2022).

Despite a noticeable fruit drop in early June (Figure S2), initial fruit set (after 4 weeks) and final fruit set (after 12 weeks) were correlated (Spearman rank correlation: *ρ* = .29, *p* < .001). Fruit set in June (after 8 weeks) and final fruit set (*ρ* = .95, *p* < .001) were highly correlated. Therefore, we used only the final fruit set in subsequent analysis. Effects of treatment (insect exclusion, open/insect pollinated and hand pollinated) on final fruit set were compared with a linear mixed effect model (LMM), with pollination treatment as predictor and site as random factor, using the *lme4* package. We also compared differences in fruit weight between treatments using an LMM, again with orchard as a random factor. To test for differences between treatment groups, a Tukey post hoc comparison was used (R package *multcomp*; Hothorn et al., 2008).

*Osmia* abundance was highly correlated with the abundance of all non‐*Apis* bees (Spearman rank correlation: *ρ* = .70, *p* < .001) in our study. Therefore, we used *Osmia* abundance in subsequent analyses. To investigate whether *Osmia* and honey bees synergistically enhanced sweet cherry fruit set (treatment O), we included the interaction between *Osmia* abundance × honey bee abundance as a fixed factor in an LMM, with orchard as a random factor.

## 3 RESULTS

### 3.1 The effect of bee management on bee abundance

A total of 10,021 flower visits were counted on sweet cherry blossoms, of which honey bees (*A. mellifera*) represented 70.2%, mason bees 15.6%, bumble bees 3.1%, other bees 6.7% and the other visitors (flies, butterflies, ants and beetles) 4.4%. The relative abundance of mason bees per site and transect walk ranged from 0% to 94.1% while relative honey bee abundance ranged from 0% to 97.1%. Fourteen of 17 farmers (82%) used honey bee hives, which ranged in density from 1.3 to 17.0 hives per hectare (Table S1). Thirteen of 17 farmers (76%) installed nesting material, mainly for mason bees, in their orchards. Nesting material density ranged from 0.1 to 8.6 boxes per hectare (Table S1). Honey bee and mason bee nesting box stocking rates were not significantly correlated to each other (Spearman rank correlation; *ρ* = −.118; *p* = .653; Figure S3).

**FIGURE 2:**
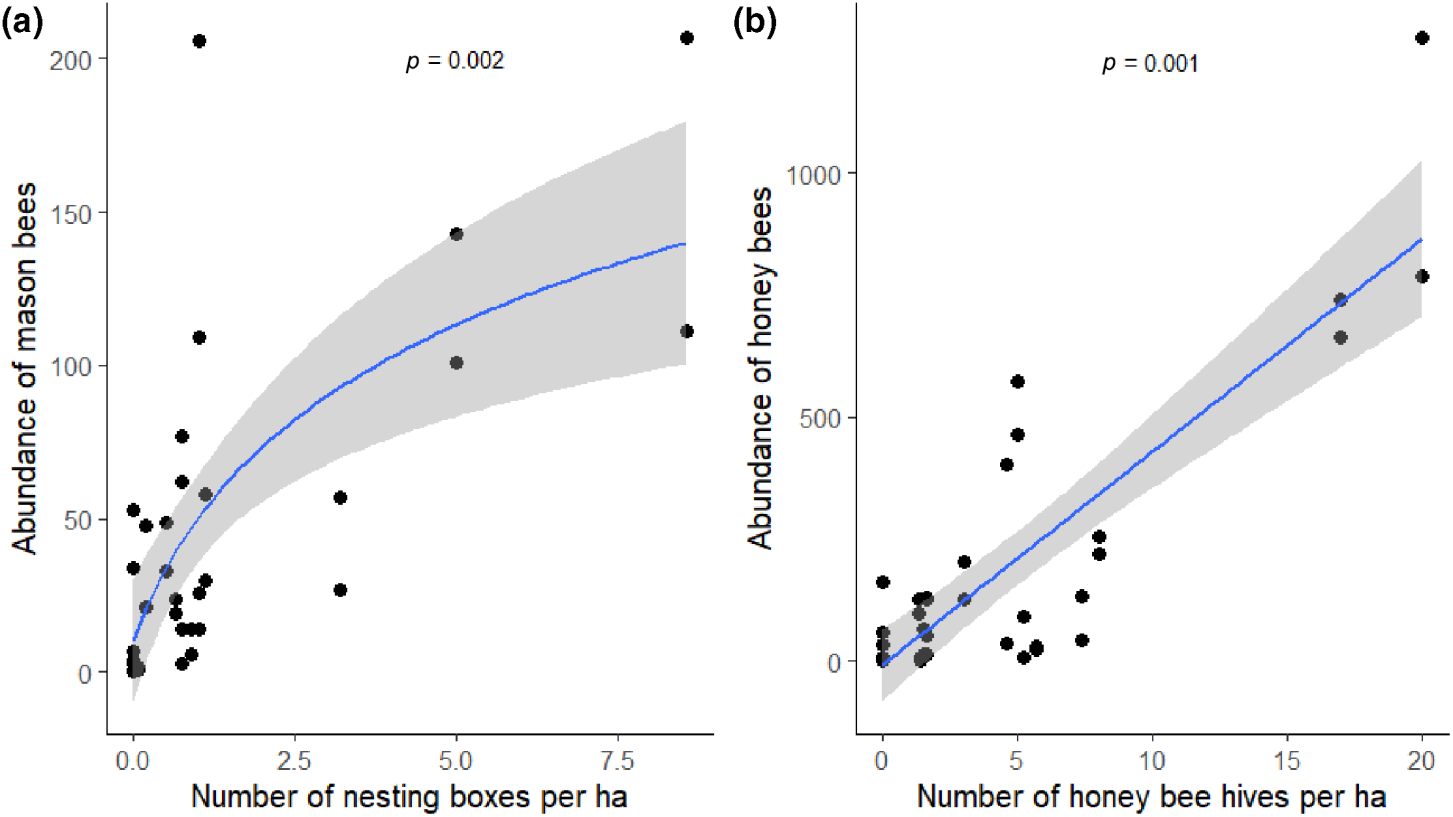
Number of mason bees (a) and honey bees (b) observed in orchards during transect walks in relation to the amount of mason bee nesting material or the number of honey bee hives per ha, respectively. The blue lines correspond to the predicted relationships (GLMM) with, in dark grey, the 95% confidence intervals.

Pollinator management had a clear effect on bee abundance. With an increasing quantity of nesting material provided, the number of observed mason bees increased, reaching an asymptote (Figure 2a; GLMM; *Z*_32_ = 3.079, *p* = .002); the non‐linear model provided the best fit to the data (smallest value of AICc, Table S2). Honey bee abundance and temperature did not affect the abundance of mason bees (GLMM; *Z*_32_ = −0.012, *p* = .464; *Z*_32_ = −1.076, *p* = .282; respectively). Honey bee abundance increased with the number of hives per ha (linear model; Figure 2b; GLMM; *Z*_32_ = 3.275, *p* = .001, Table S2) and with ambient temperature (GLMM; *Z*_32_ = 20.016, *p* < .001). Mason bees in the trap nests collected mainly *Prunus* pollen, most likely from cherry trees during full bloom (Figure S4).

### 3.2 Sweet cherry fruit set and the effect of bee flower‐visitation rates

The final fruit set of bagged flowers (insect exclusion, treatment B) varied between 0% and 6% (mean: 2%, Figures S5 and S6). These data demonstrate the need for sweet cherry flowers to be pollinated by insects to successfully set fruit. The average fruit set of sweet cherries at harvest (treatment O) varied between 9% and 69% (mean: 28%) across orchards. Hand pollination (treatment H) varied between 7% and 68% (mean: 39%), though was generally higher than treatment O (Figure S6). Statistical analysis demonstrated that pollination treatments were significantly different at harvest (Tukey post‐hoc, *p* < .001), reinforcing the strong positive impact of insect pollination on fruit set (insect exclusion vs. insect pollination) and highlighting pollination limitation in our study orchards (insect pollination vs. hand pollination; Figures S5 and S6). Fruit weight (g) per harvested fruit did not differ between treatments (Tukey post‐hoc, *p* > .426; Figure S7).

**FIGURE 3:**
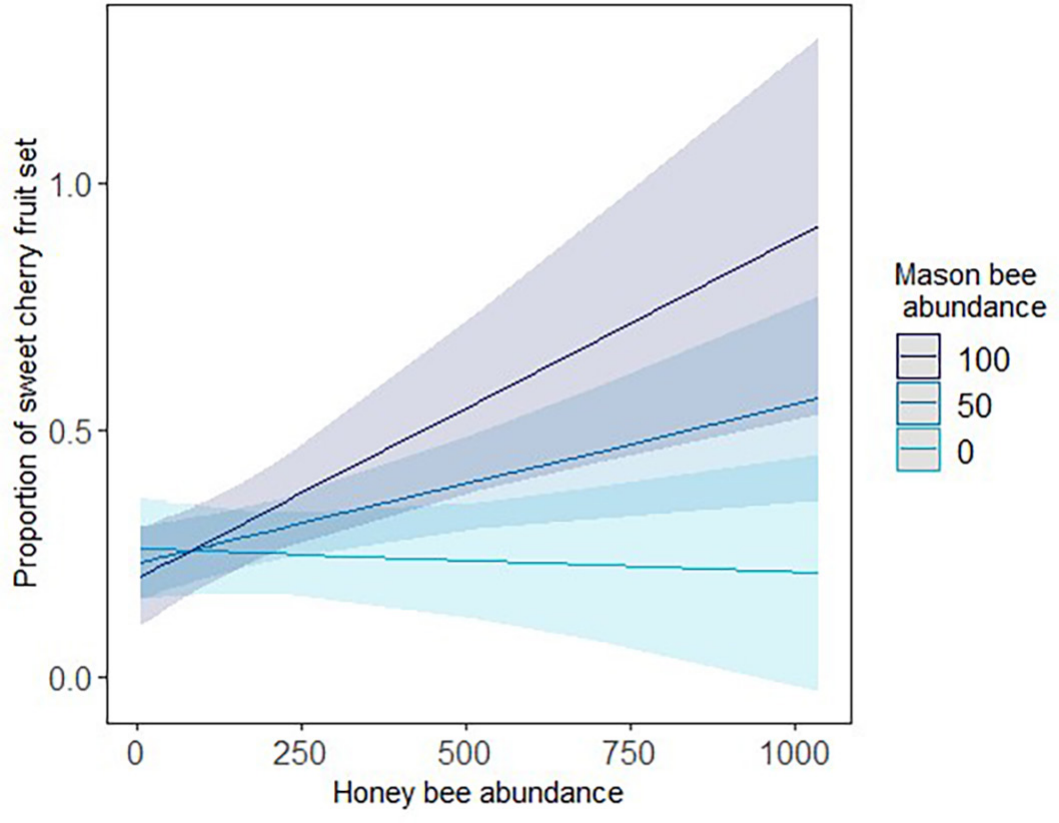
Predicted interaction effect of mason bee and honey bee abundances on the proportion of sweet cherry flowers that set fruit (treatment O), estimated from an LMM. Effects of increasing honey bee abundance are plotted for different mason bee abundances: no mason bees present (bright blue line), 50 mason bees per transect walk (blue line) and 100 mason bees per transect walk (dark blue line). Lines correspond to predicted relationships and shaded areas to 95% confidence intervals derived from the linear mixed effect model.

We found that honey bee and mason bee abundances interactively increased open final fruit set in sweet cherry orchards (LMM, *t* = 2.77, *p* = 0.016; Figure 3). In the absence of *Osmia* (bright blue line) sweet cherry fruit set (treatment O) did not improve as honey bee numbers increased. In orchards with medium mason bee abundance, sweet cherry fruit set increased with rising honey bee numbers (blue line). This trend was even stronger in orchards with the highest mason bee abundance (dark blue line). The inverse was also supported; by only increasing mason bee abundance at low honey bee abundance, sweet cherry fruit set remained low (left side Figure 3).

## 4 DISCUSSION

We found that the abundance of sweet cherry flower‐visiting mason bees can be enhanced by providing nesting material. This relationship was, however, not linear but asymptotic, which is in contrast to a linear increase for honey bees with hive stocking rates. Mason bees synergistically with honey bees enhanced sweet cherry fruit set, a crop that is highly dependent on insect pollination.

### 4.1 Enhanced pollinator abundance through on‐farm measures

Here we demonstrate that, by providing nesting material, farmers can enhance mason bee abundance on sweet cherry blossoms. Notably, the relationship between nesting material per ha and *Osmia* abundance as sweet cherry flower visitors was non‐linear but reached an asymptote. One reason might be a result of increasing rates of brood cell parasitism by kleptoparasites and other pests with increasing density of mason bees (Groulx & Forrest, 2018) that is, density‐dependent parasitism. By providing an excess of nesting material, brood parasitism rates may increase and therefore the numbers of foraging bees may be reduced below that expected for the total amount of nesting material made available. Alternatively, at five or more nest boxes per ha, resources for mason bees may be saturated even in a mass‐flowering crop (Eeraerts et al., 2021), especially when honey bees are employed simultaneously (Herbertsson et al., 2016; Lindström et al., 2016; Weekers et al., 2022).

Our results show that the steepest increase in flower‐visiting mason bees can be observed when transiting from zero to two nesting boxes per ha (see Figure 2). We therefore recommend farmers to install two nesting boxes per ha, each containing approximately 500 nesting tubes, to maximise sweet cherry pollination. Cocoons of mason bees do not have to be purchased and imported to an orchard as previous studies have shown that local *Osmia* populations readily colonise new nesting material, even in agricultural landscapes, and increase yearly in numbers with an intrinsic rate of increase (*r*) between 1.3 and 2.8 (Bosch et al., 2021; Gruber et al., 2011; Steffan‐Dewenter & Schiele, 2008). Promoting local mason bee populations without the need to purchase commercially reared cocoons is, therefore, possible and should be preferred, as the import and trade of bees can have negative impacts on wild bee populations (Aizen et al., 2020; Osterman, Aizen, et al., 2021; Pirk et al., 2017).

Honey bee abundance on flowers of a target crop can also be enhanced by employing honey bee hives. In contrast to mason bees, we found that the relationship between honey bees on flowers and hive stocking rate was linear; the more honey bee hives provided, the more honey bees observed during transect walks. This an interesting observation, as other studies in blueberry and pumpkin fields have found no effect of stocking rates on honey bee visitation rates (Mallinger et al., 2021; Petersen et al., 2013). One explanation might be that sweet cherry is an attractive food resource for honey bees and they are not as attracted by co‐flowering crops or wild plants (Bänsch et al., 2021; Osterman, Theodorou, et al., 2021), while this might not be true for blueberries and pumpkin.

### 4.2 Mason bees as reliable flower visitors of sweet cherry

The preference of *Osmia* bees for fruit trees (Torchio, 1976; Vaudo et al., 2020; Vicens & Bosch, 2000b), specifically of *O. cornuta* (Eeraerts et al., 2021), is in line with our results of mason bees collecting mainly *Prunus* pollen during cherry full bloom. Also, the stable visitation rates by *O. cornuta* throughout the day compared to honey bees, whose visitation of Rosaceae flowers peaks in the afternoon (Vicens & Bosch, 2000a), support our findings of only honey bee numbers on flowers, but not mason bee numbers, being positively influenced by temperature. During inclement weather conditions, mason bees can be reliant pollinators of sweet cherry flowers (Vicens & Bosch, 2000a) as they fly throughout the day, even during colder temperatures. This, in combination with the higher efficiency of mason bees as cherry pollinators compared to bumble bees and honey bees (Eeraerts, Vanderhaegen, et al., 2019), makes them an optimal bee to promote for the pollination of sweet cherry.

### 4.3 The effect of mason bees on sweet cherry fruit set

We found that mason bees interactively with honey bees increased fruit set of sweet cherry. When mason bee abundance was low, sweet cherry fruit set did not increase with increasing honey bee abundance. A positive relationship was only seen when mason bee abundance increased simultaneously. This suggests that non‐*Apis* bees, including managed and wild bee species, could facilitate pollination services by honey bees in crops or cultivars, especially those dependent on cross pollination. For several flowering crops, honey bees have been found to have an increased visitation rate, higher probability of row changes and higher single visit efficiency with increasing non‐*Apis* bees (sunflower: Greenleaf & Kremen, 2006; almond: Brittain et al., 2013; apple: Sapir et al., 2017; cherry: Eeraerts et al., 2019b). Through a change in the behaviour of the dominant flower‐visiting species, interspecific interactions among pollinator species were found to synergistically enhance almond nut set in experimental cages (Brittain et al., 2013), supporting the evidence that pollinator diversity contributes to crop pollination (Hünicken et al., 2022; Radzevičiūtė et al., 2021; Woodcock et al., 2019). However, in our study we cannot identify the mechanism by which mason bees and honey bees interacted to enhance sweet cherry pollination. To do so, additional information would be needed on the behaviour of the bees along with the single visit pollination efficiency (sensu Spears, 1983) of honey bees along a gradient of mason bee abundance. Another explanation for the positive interactive impact of honey bees and mason bees on sweet cherry pollination could be that mason bees and honey bees occupy different functional foraging niches on cherry flowers and therefore jointly enhance its pollination (Hünicken et al., 2022).

Surprisingly, when mason bee abundance was high but honey bee abundance low, fruit set was also low; an effect of mason bees on sweet cherry pollination was only found with increased honey bee abundance. Mason bees have been shown to be effective pollinators of sweet cherry (Eeraerts, Vanderhaegen, et al., 2019) and other fruit trees (Monzón et al., 2004). However, in commercial orchards, as employed in our study, honey bees were the predominant flower visitors (average of 70% in our study), while mason bee abundance seems to reach a saturation point, at which additional nesting material did not increase mason bee abundance. Only relying on mason bee populations might not result in a stable and sufficient yield. This is supported by findings by Hünicken et al. (2022), who detected a consistent lower productivity of crops pollinated by mason bees compared to those pollinated by honey bees.

To maximise sweet cherry pollination, we recommend a combination of employing honey bees as well as habitat enhancement measures for wild bees, especially in intensively managed agricultural landscapes in which wild bees are rare. However, honey bee hive densities should be moderate to avoid the deleterious effects of high honey bee densities on wild bee populations (Herbertsson et al., 2016; Lindström et al., 2016). Reliance of crop pollination on one pollinator species alone might bear risks and result in limited fruit set. Future studies should investigate optimal management practices (e.g. honey bee hive density) applied to local conditions, that is density of wild bees, landscape variables, crop type and crop cultivar, and disseminate them to farmers to ensure stable fruit and seed set.

### 4.4 Risks associated with managing pollinators

Despite the benefits of managing pollinators in enhancing crop yields, risks associated with their management should also be taken into consideration (Russo et al., 2021). The promotion of local wild bee populations by providing nesting material is most likely not harmful and should be preferred over the rearing and trade in managed species, even of native solitary bees, as local adapted populations could be genetically swamped by managed bees (Russo et al., 2021). Nevertheless, habitat enhancement (e.g. increasing the quality and quantity of floral resources, protecting and restoring semi‐natural habitats) for native pollinators in and around crop fields should be given priority to enhance the diversity of pollinators (Osterman, Aizen, et al., 2021). For instance, by providing co‐flowering plants or managing orchards organically, cherry pollinator richness can be enhanced (Gilpin, O'Brien, et al., 2022; Mateos‐Fierro et al., 2023; Rosas‐Ramos et al., 2020). Notably, measures promoting pollinators should be tailored to geographical regions and local conditions, as pollinator communities differ between regions (Dar et al., 2018). Importantly, providing nesting material might be beneficial for other sweet cherry pollinators in other regions (Gilpin, Brettell, et al., 2022).

## 5 CONCLUSIONS

Our study demonstrates that the presence of nesting material can enhance mason bees as flower visitors in cherry orchards. Fruit set of sweet cherry, a highly pollinator‐dependent crop, was synergistically increased by honey bee and mason bee abundances. As other studies have similarly highlighted the facilitative component of non‐*Apis* bees on the performance of honey bees, we encourage farmers to implement measures to protect diverse wild pollinator communities in orchards. Combining several measures to promote pollination services represents a sustainable way to ensure resilient crop pollination.

## AUTHOR CONTRIBUTIONS

**Julia Osterman:** Conceptualization (lead); data curation (lead); formal analysis (lead); investigation (lead); methodology (lead); project administration (lead); visualization (lead); writing – original draft (lead); writing – review and editing (lead). **Frances Benton:** Data curation (supporting); investigation (supporting); methodology (supporting); writing – review and editing (supporting). **Sara Hellström:** Investigation (supporting). **Meike Luderer‐Pflimpfl:** Conceptualization (supporting); data curation (supporting); formal analysis (supporting); investigation (supporting); methodology (supporting); project administration (supporting); writing – review and editing (supporting). **Ann‐Kathrin Pöpel‐Eisenbrandt:** Investigation (supporting); writing – review and editing (supporting). **Bilyana Stoykova Wild:** Data curation (supporting); investigation (supporting); methodology (supporting); project administration (supporting); writing – review and editing (supporting). **Panagiotis Theodorou:** Formal analysis (supporting); writing – review and editing (supporting). **Christin Ulbricht:** Conceptualization (supporting); investigation (supporting); methodology (supporting); project administration (supporting); writing – review and editing (supporting). **Robert J. Paxton:** Conceptualization (supporting); formal analysis (supporting); investigation (supporting); methodology (supporting); supervision (supporting); writing – original draft (supporting); writing – review and editing (supporting).

## FUNDING INFORMATION

J.O. was supported by the ESCALATE graduate school of the UFZ and the Carl Trygger Foundation (CTS 21:1757). M.L.‐P. and A.‐K.P.‐E. were founded by The Thüringer Ministeriums für Infrastruktur und Landwirtschaft (TMIL) with the project number 2019 LFE 0007. F.B and B.S. were financed by the ERASMUS exchange programme of the European Union.

## CONFLICT OF INTEREST STATEMENT

None.

## Supporting information


Appendix S1.

## Data Availability

The data are available via the Dryad Digital Repository: https://doi.org/10.5061/dryad.3xsj3txn0.
