# Supplementary material for: Mason bees and honey bees synergistically enhance fruit set in sweet cherry orchards
Source: Ecol Evol. 2023 Jul 9;13(7):e10289. doi: 10.1002/ece3.10289 (PMC10329911; doi:10.1002/ece3.10289)
Supplement: Supplementary file 1 — Appendix S1. [file ECE3-13-e10289-s001.docx]

**Mason bees and honey bees synergistically enhance fruit set in sweet cherry orchards**

Supplementary Materials

Osterman *et al*.

Correspondence: jul.osterman@gmail.com

This PDF file includes:

Supplementary Tables S.1 and S.2

Supplementary Figures S.1 – S.7

**Supplementary Tables**

**Table S.1.** Description of the study sites

| **Number** | **Orchard** | **Orchard management*** |  | | **Bee management** | | **Coordinates**** | | **Pollinizer cultivar** | **Orchard size in ha** | **Area of cherry cultivation in ha** |
| --- | --- | --- | --- | --- | --- | --- | --- | --- | --- | --- | --- |
|  |  |  | **Mason bees** | | | **Honey bees** | **Latitude** | **Longitude** |  |  |  |
|  |  |  | **Boxes/ha** | **Initial year of mason bee management** | | **hives/ha** |  |  |  |  |  |
| 1 | Aseleben | Commercial, IPM | 0.65 | 2020, released cocoons | | 1.61 | 51.572140 | 11.767439 | ‘Rubin’ | 6.22 | 6.22 |
| 2 | Beesenstedt | Commercial, IPM | 0.06 | 2017 | | 1.65 | 51.572503 | 11.732081 | ‘Sylvia’ | 18.00 | 8.50 |
| 3 | Beyernaumburg | Commercial, IPM | 0 | NA | | 0 | 51.470662 | 11.414594 | ‘Henriette’ | 6.00 | 6.00 |
| 4 | Doellstaedt | Commercial, IPM | 0.88 | 2013 | | 1.48 | 51.064875 | 10.782511 | ‘Rubin’ | 6.75 | 6.75 |
| 5 | Eisleben | Commercial, IPM | 1.00 | 2005 | | 5.20 | 51.507664 | 11.565084 | ‘Henriette’ | 3.00 | 2.50 |
| 6 | Erfurt | Experimental, IPM | 8.57 | 2002 | | 0 | 50.992652 | 11.052581 | ‘Rubin’ | 1.40 | 0.84 |
| 7 | Gatterstaedt | Commercial, IPM | 0 | NA | | 8.00 | 51.399474 | 11.523638 | ‘Rubin’ | 12.00 | 2.00 |
| 8 | Grossfahner | Commercial, IPM | 1.10 | 2013 | | 7.38 | 51.053088 | 10.815892 | ‘Rubin’ | 2.71 | 2.71 |
| 9 | Hoehnstedt | Commercial, IPM | 1.00 | 2014 | | 5.70 | 51.499598 | 11.757832 | ‘Sam’ | 130.00 | 7.00 |
| 10 | Johannashall | Commercial, IPM | 0.75 | 2020, released cocoons | | 3.00 | 51.572140 | 11.767439 | ‘Skeena’ | 4.00 | 4.00 |
| 11 | Kleinfahner | Commercial, IPM | 0.20 | 2013 | | 16.95 | 51.036593 | 10.854890 | ‘Rubin’ | 5.12 | 5.12 |
| 12 | Kindelbrueck 1 | Commercial, IPM | 0.75 | 2008 | | 1.40 | 51.254971 | 11.100536 | ‘Sam’ | 20.00 | 20.00 |
| 13 | Kindelbrueck 2 | Commercial, IPM | 0.50 | 2008 | | 4.60 | 51.248409 | 11.057025 | ‘Sam’ | 36.00 | 36.00 |
| 14 | Ploessnitz | Commercial, Organic | 0 | NA | | 20.00 | 51.539550 | 12.060131 | ‘Kordia’ | 15.00 | 2.00 |
| 15 | Quedingburg | Experimental, IPM | 3.20 | 2014 | | 1.33 | 51.813164 | 11.199462 | ‘Fertar’ | 7.50 | 0.16 |
| 16 | Querfurt | Commercial, IPM | 5.00 | 2015 | | 5.00 | 51.370171 | 11.588193 | ‘Rubin’ | 11.00 | 2.00 |
| 17 | Spoeren | Commercial, IPM | 0 | NA | | 0 | 51.606711 | 12.113216 | ‘Canada Giant’ | 7.50 | 0.50 |

* IPM: Integrated Pest management; Experimental orchards are owned by research institutions for conducting research experiments but are managed as commercial orchards.

** Coordination system: Mercator projection WGS84 in Decimal Degrees

**Table S.2.** Results of model selection of the bee abundance analysis based on the AICc criterion

| **Model** | | **Intercept** | **Effect measure** | **Estimate** | **Z** | **P*** | **AICc**** |
| --- | --- | --- | --- | --- | --- | --- | --- |
| *Osmia* abundance | Linear model | 2.542 | Wild bee nesting/ha | 0.587 | 2.478 | **0.013** | 333.83 |
| on flowers |  |  | Temperature | -0.012 | -0.723 | 0.470 |  |
|  | **Log (x+1) model** | 2.182 | Log(wild bee nesting/ha +1) | 1.775 | 3.079 | **0.002** | **331.44** |
|  |  |  | Temperature | -0.012 | -0.732 | 0.464 |  |
|  |  |  |  |  |  |  |  |
| Honey bee abundance | **Linear model** | 2.102 | Honey bee hives/ha | 0.141 | 3.275 | **0.001** | **778.67** |
| on flowers |  |  | Temperature | 0.121 | 20.016 | **<0.001** |  |
|  | Log (x+1) model | 1.675 | Log(hives/ha +1) | 0.790 | 2.602 | **0.009** | 781.21 |
|  |  |  | Temperature | 0.121 | 20.024 | **<0.001** |  |

*** P values < 0.05 are highlighted in bold.

** Lowest AICc values are highlighted in bold


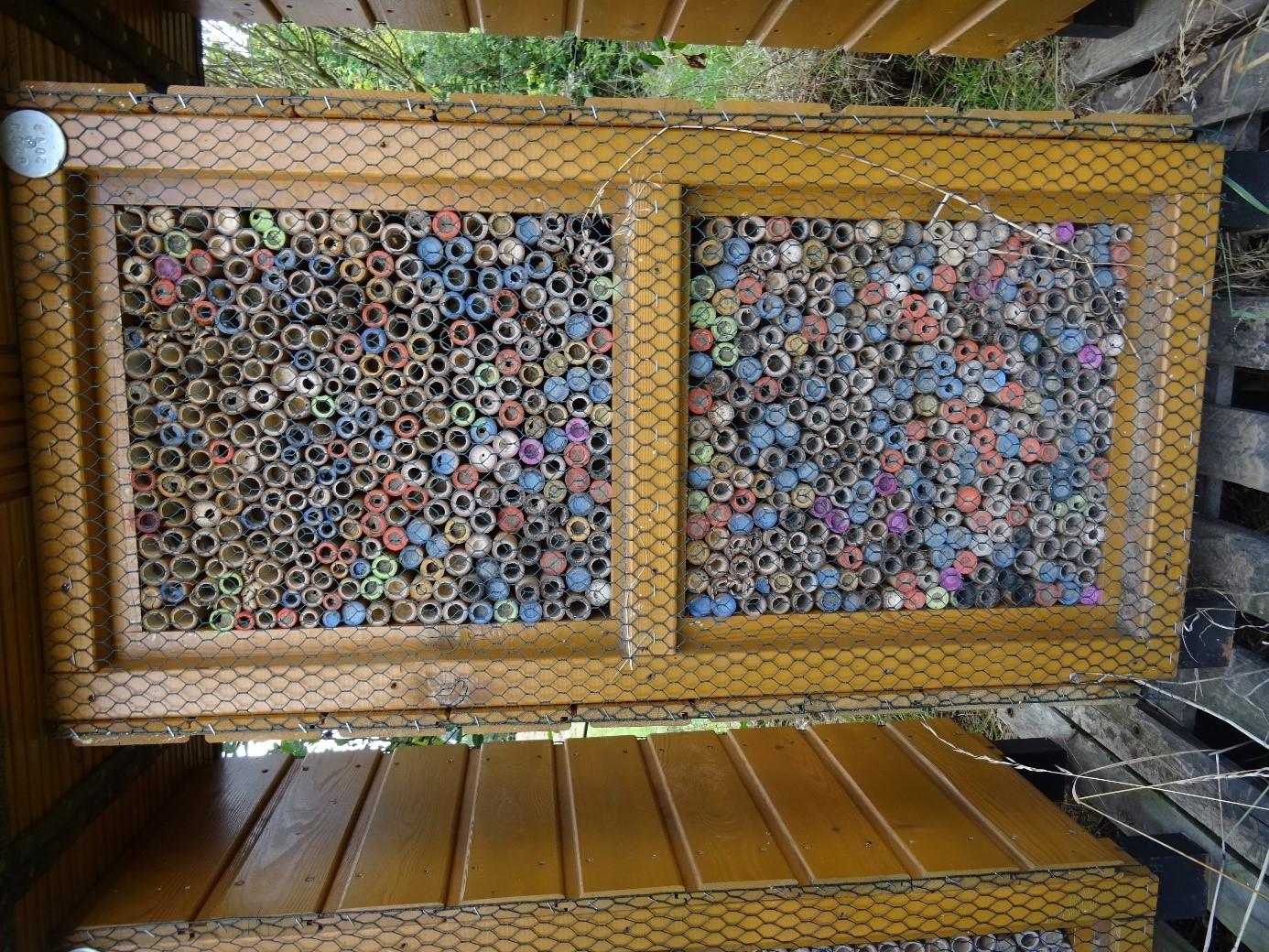


Figure S.1: Example of artificial mason bee nesting material used in sweet cherry orchards.


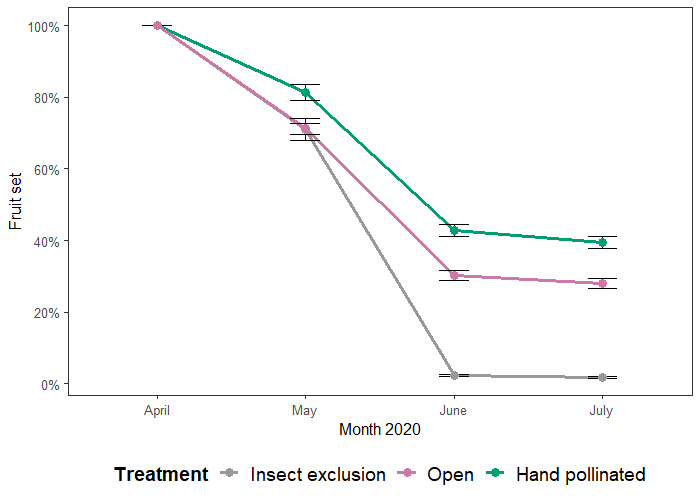


Figure S.2: Sweet cherry fruit set (in percentage; mean ± S.E) measured at three time points over time for three treatments.


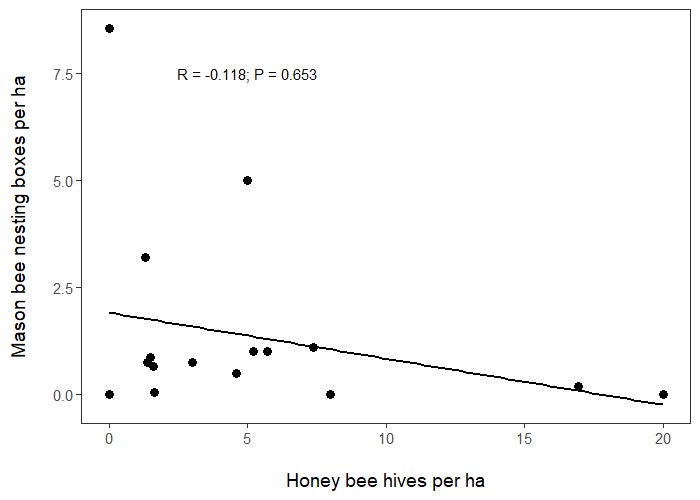
Figure S.3: Spearman rank correlation between mason bee nesting boxes per ha and honey bee hives per ha.


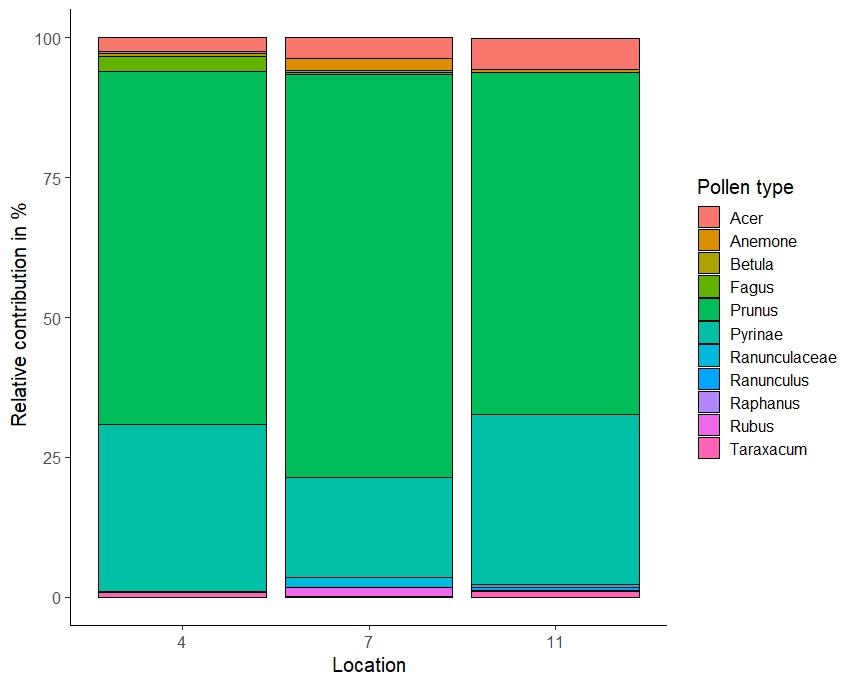


Figure S.4: Pollen types identified in trap nests (two pools of pollen per location) at three locations. Pollen listed as Pyrinae was most likely *Prunus* but could not be confirmed as such.


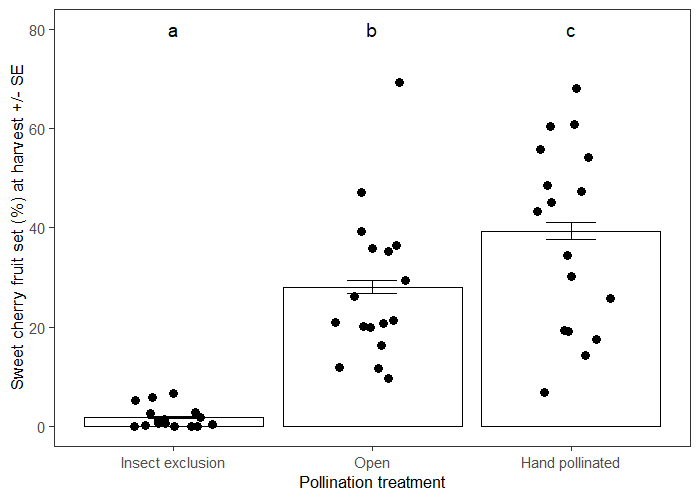


Figure S.5: Mean sweet cherry fruit set (%) for experimental pollination treatments ± S.E. Different letters indicate significant differences (LMM; Tukey post hoc, *P* < 0.05). Dots represent fruit set per orchard.


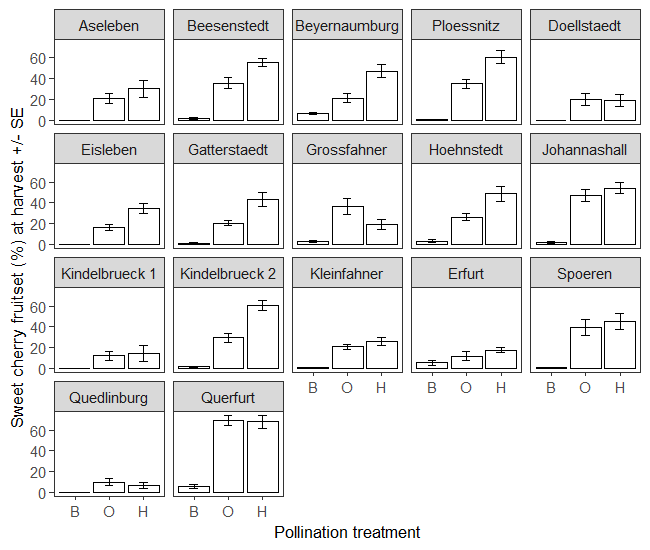


Figure S.6: Mean sweet cherry fruit set (%) for experimental pollination treatments ± S.E. (B = Bagged/Insect exclusion; O = Open pollination; H = Hand pollination) per study site.


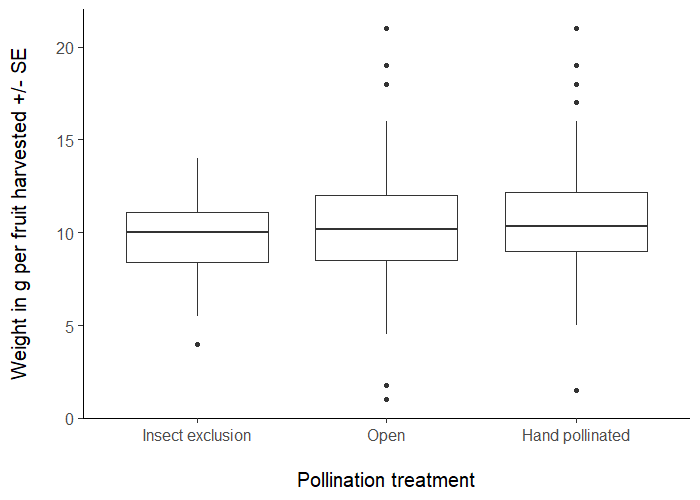


Figure S.7: Weight in g per ripe cherry for the pollination treatments. Horizontal line in the box give the median, whiskers and outlier dots indicate the 25th–75th percentiles and minimum–maximum, respectively.
